# Decision-Making Is in the Making! Aspects of Decision-Making in the Area of Assistive and Welfare Technology—A Qualitative Study

**DOI:** 10.3390/ijerph18084028

**Published:** 2021-04-12

**Authors:** Katarina Baudin, Angelina Sundström, Johan Borg, Christine Gustafsson

**Affiliations:** 1 School of Health, Care and Social Welfare, Mälardalen University, SE-63105 Eskilstuna, Sweden; christine.gustafsson@mdh.se; 2 School of Innovation, Design and Engineering, Mälardalen University, SE-63105 Eskilstuna, Sweden; angelina.sundstrom@mdh.se; 3 Department of Health and Welfare, Dalarna University, SE-79188 Falun, Sweden; jog@du.se

**Keywords:** assistive technology, welfare technology, decision-making, managers, assistive technology organizations, older adults, thematic analysis

## Abstract

Assistive and welfare technology (AT/WT) has been introduced as a way of facing an ageing population and providing support for older adults in their daily lives. There is much research concerning the assessment and recommendation of AT/WT to individual end-users. However, few studies have explored AT/WT decision-making from a managerial perspective. This study explores what aspects influence decision-making in assistive technology organizations concerning new technology procurements. The study is based on interviews with 24 managers engaged in assistive technology organizations, representing 13 of 21 regions in Sweden. The interview data consisted of the participants’ experiences deciding on AT/WT procurement. A reflexive inductive thematic analysis was used to identify aspects that influenced decision-making. The main findings show that decision-making is in the making, meaning that decision-making is a constant on-going managerial process. Furthermore, the findings show that managers experience uncertainty in the decision-making, sometimes make ad hoc decisions and request an evidence-based, person-centred approach to improve decision-making. The study concludes that supportive, technology, patient, and knowledge aspects influence managers’ decisions.

## 1. Introduction

Life expectancy is increasing in many countries, which leads to an ageing population. As the ageing population grows, the resulting demographic changes will increase the need for health, care, and rehabilitation services [1,2]. An option favoured by policymakers and older adults is to support individuals to remain in their own homes longer and avoid residential care, also known as “ageing in place” [3]. Older adults may age in place with the support of assistive technology (AT) and welfare technology (WT), which have thus been introduced as possible solutions to meet part of the demographic challenges [4].

The concept of AT embraces universally designed, every day and mainstream products, remote technologies, monitoring and sensing devices, and a variety of information and telecommunication technologies [5]. As AT is designed for people with diverse functioning abilities, many low- and high-technology devices are available [6], and the usage of AT has been suggested to, for example, assist people and treat different chronic diseases [7]. In the Scandinavian context, where this study was conducted, WT is a well-known concept. However, it is complex and has evolved from merely an application to include applications, administration services, and systems [8]. According to the National Board of Health and Welfare in Sweden [9], “WT is a digital technology with the purpose of maintaining or increasing security, activity, participation or independence for a person with or elevated risk for a disability.” This definition of WT includes both technology devices and intelligent systems, and the area has expanded to address the needs caused by the demographic changes. Thus, there is an ongoing discussion about the two concepts AT and WT and their definitions, as well as potential overlaps between their definitions and usage [8,10,11,12,13]. Due to the lack of consensus in the literature, we use AT for the purpose of this paper, as AT also encompasses the types of WT included in this study. In line with prior research on AT, we argue that AT addresses the challenges of a growing older population and may support people with an elevated risk of disability in their daily lives. 

Given that financial resources in the public sector are limited [14], decision-makers in AT organizations need to make well-founded decisions about what AT to access and when and to whom to provide the technology. The decision-makers in AT organizations must acknowledge that public financial resources are used to fund health and social care. While considering that public financial resources are scarce and AT is expected to reduce the need for formal health and social care support services, long-term care, and the burden of caregivers, it is essential to acknowledge that AT is designed to support daily living, which must be understood and assessed in their context [15,16]). However, to the best of our knowledge, the previous literature on AT decision-making fails to address decision-making in AT organizations, especially aspects that influence the decisions that decision-makers (i.e., managers) make regarding specific AT provision [17,18,19,20].

Most of the prior research on AT decision-making focuses on decision-making regarding the evaluation and recommendation of AT to the individual end-user [21], and there are several reports of the processes and procedures associated with this matter [21,22,23,24]. The literature addresses this concern in terms of AT assessment, emphasizing the importance of including the user and the user’s abilities, needs, preferences, and challenges when matching AT with the user. Thus, researchers suggest that AT evaluation and recommendations need to be based on a person-centred approach [25,26,27,28,29] to acknowledge these aspects and to maximize the match between AT and the end-user. The model of human occupation (MOHO), proposed by Kielhofner [30], is an example of understanding and practicing the person-centred approach. MOHO is an occupational therapy conceptual practice model based on a dynamic systems theory and explains how the person’s performance capacities, habituation (i.e., habits, roles, patterns), and volition (i.e., motivation) interact with environmental conditions and influence the capacity to manage AT [30]. It puts forward a holistic view of the older adult, directing attention towards the medical needs and life situation of the individual AT end user [30]. 

### Aim of the Study

This study sought to explore aspects influencing decision-making in assistive technology organizations concerning new technology procurement. Furthermore, the study aimed to contribute to the knowledge on AT, specifically on AT decision-making. This was achieved by exploring the area of AT decision-making, focusing on the managerial level in AT organizations in Sweden. 

The remainder of the paper is structured as follows. In Section 2, the materials and methods used in this study are outlined. Section 3 presents the empirical results, which are then discussed in Section 4 along with directions for future research. The conclusions are presented in Section 5.

## 2. Materials and Methods

This study adopted a qualitative research methodology using a qualitative descriptive design. This methodology is beneficial when not much is known about a specific topic or phenomenon and when there is a desire to gain a deeper understanding [31]. There are few studies on AT decision-making in AT organizations; therefore, the qualitative descriptive design was considered as an appropriate design to use. 

The study focuses on exploring decision-making regarding AT and AT organizations located in Sweden. To gain insights on this topic, the National Network of Assistive Technology Managers was contacted. 

### 2.1. Participants

The selection of participants was based on stratified purposive sampling [32]. A group of eight manager representatives, called the reference group, from the National Network of Assistive Technology Managers recommended 30 managers for the study. The criterion for participation was managers with decision-making power in the discussion about adding AT assortment to the purchase or procurement of new AT in their respective assistive technology organizations. Advantages of this sampling method were that the final sample consisted of participants who could contribute with their experience and knowledge and that the manager representatives could confirm the participants’ involvement in the decision-making regarding AT in AT organizations. 

A total of 20 managers expressed interest in participating in the study. This interest resulted in a small snowball effect [33], which provided the study with four additional participants. Thus, there were 24 participants, representing 13 of the 21 regions in Sweden. Those who did not participate had either retired, changed position, or did not have time to participate. Three of the 20 managers who were contacted suggested other persons that could be interviewed instead. 

The participants were initially contacted by email, where the project was described, and a request was made for them to participate in the study. They were given written information about the interview, telling them that it would be recorded, and how the data would be stored. This information was given to all participants before their interview. The majority of the contacted managers were positive about participating in and contributing to the study, suggesting that this was an important area that needed to be explored. When they confirmed their participation, a day and time for a telephone interview were booked.

### 2.2. Data Collection 

Based on Charlton [34], a semistructured interview guide was designed. As a first step, a pilot test was conducted with two managers within an AT organization, who were interviewed about their organization and work. Based on the pilot test experiences, preliminary semistructured question areas were created. Additionally, the GATE domains—policy, assessment, procurement, technology, environment, usability, sustainability, and rights [35]—provided inspiration for the areas of these preliminary semistructured questions. The semistructured question topics were then sent to the reference group (i.e., the National Network of Assistive Technology Managers). This was done to obtain feedback on the topics and to see if other vital topics were missing. After receiving the responses of the reference group, the semistructured interviews were finalised, containing eight domains of questions and subquestions. The topic areas in the final interview guide were (1) organization, (2) policy, (3) AT provision, (4) AT procurement, (5) decision-making, (6) accessibility of information, and (7) AT, technology development, and (8) competence. Discussed within the topic areas 2–8, MOHO was used to understand how person-centredness is considered by managers when making AT decisions.

The interviews were conducted by phone between November 2018 and February 2019. The length of the interviews varied between 30 and 60 min, which is recommended by Kvale [36]. During the interviews, the participants also shared their local decision-making supports and guidelines for clarification and as a complement of their AT systems and organization. As suggested by Thurmond [37], these documents and decision-making supports were read multiple times and included in the thematic analysis to strengthen the validity of the findings. 

### 2.3. Analysis

**Figure 1:**
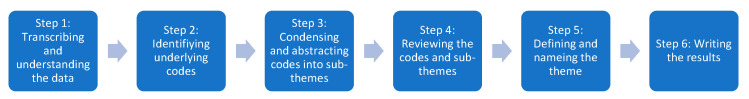
An illustration of the thematic analysis steps in this study (following Braun and Clark [39]).

The gathered information was analysed based on inductive reasoning, with themes emerging from data [38]. Following the steps in Braun and Clarke [39], a reflexive inductive thematic analysis was applied to the answers of the semistructured questions, and codes, subthemes, and an overall theme emerged (see Figure 1). To strengthen the trustworthiness of the analysis process, the authors’ preunderstanding was taken into consideration by using critical reflection and slowing down the process of understanding. The researchers varied in terms of professional background (industrial economics and management, nurse, and occupational therapist), experience with the target group, and qualitative research method. Further, one of the authors (KB) performed the interviews and thus had an insider’s perspective regarding data collection, while the other three authors, who had not collected the data, had an outsider’s perspective.

The analysis included the step of transcribing the interview data. The transcribed interviews were read several times to achieve an overall understanding of the data. The analysis processes then continued by identifying the underlying codes, followed by condensing and abstracting codes into subthemes. Similarities and differences that guided the organization of subthemes were then recognised on a descriptive level. Lastly, a theme, which was the common thread including the various subthemes and codes related to the study’s aim, was identified. The analysis process of identifying codes, subthemes, and the overall theme was supported by the software program NVivo 11. An iterative process was used, and the focus switched between the whole and parts of the data during the analysis. The themes, subthemes, and codes that emerged were discussed within the author group until a consensus was reached. After the analysis of 12 interviews, data saturation occurred, which is in line with Guests’ [40] discussion about data saturation. The analysis of the rest of the interviews confirmed the emergent subthemes from these 12 interviews. Hence, this study ensured the trustworthiness and authenticity of the information, in line with the suggestions of Berg [41] and Polit and Beck [33]. Inspired by Thurmond’s discussion on the importance of triangulation [37], a document review regarding local directives, regulations, and the different organizations’ websites was carried out, and the documents were read several times. Additionally, another scholar with experience in qualitative data analysis who was not involved in the present study verified the accuracy of the analysis and the interpretation of the themes.

### 2.4. Research Ethics 

This study did not include any personal or sensitive information that required ethical approval under the standards of the Swedish Research Council. The study followed the guidelines for research ethics issued by the Swedish Research Council [42].

## 3. Results

The data revealed an overarching theme,” Decision-making is in the making”, referring to the ongoing work and challenges the decision-makers face related to the political, social, and legal factors in society. The four subthemes that emerged from the analysis were supportive aspects, technology aspects, patient aspects, and knowledge aspects (see Table 1).

### 3.1. Supportive Aspects 

The subtheme Supportive aspects included three codes: policies and guidelines, development initiatives, and diverse management. The first code, policies and guidelines, focuses on the support that the managers have in their decision-making, such as formal written documents (e.g., policies and guidelines) that they need to consider, and which regulate their decisions.

The next code, development initiatives, focused on the managers’ initiatives to develop work procedures and guidelines when they felt a lack of support. The third code, diverse management, also involved the existing differences in work procedures among different AT organizations. Each code will be further discussed below. 

#### 3.1.1. Policies and Guidelines 

The different policies and guidelines the participants used depend on the AT organizations they worked for, which in turn were under the influence of the regional or municipal AT organizations they belong to. All the participants mentioned that they have policies and guidelines that supported them in their decisions when procuring large-volume assortments, such as ordinary wheelchairs, walkers, shower seats, or other similar AT. However, regarding new specific innovative technology brought up on the agenda by older adults, relatives, or prescribers, some of the managers make ad hoc decisions.

*In special cases, one single assistive technology to a few individual persons […] We have only CE marked [technologies], and it must be within the responsibility municipality. We have made the distribution with the ISO codes. When the technology is classified as a medically classified product, the product needs to meet a standard and fulfil the requirements [before being considered]. For example, the Mollii suite has an ISO code. We also have a decision support model [from nearby University], a prioritization’s model. We use this as a basis for decisions to make a priority at the individual level […]. (Participant 11)*

#### 3.1.2. Development Initiatives 

Although there were policies and guides that regulate the decision-making and support of the managers, the participants reported that parts of these formal written documents were not detailed or supportive enough. For example, the managers stated that the agreements between the region and the municipality were unclear and that they felt there was a lack of supportive guidelines and procedures for decision-making. The participants stressed that their feelings of inadequate support led them to take initiatives to develop their own supportive guidelines and procedures.

*We have much that regulates and give directives. We have a care guide (sv. Vårdguiden) and in our municipality also the aid guide (sv. hjälpmedelsguiden). We also have local guidelines and procedures about technical safety. It is clear when I talk to the prescribers that not much has developed since I worked as an occupational therapist. Is it we or the region that should do this? For example, in the agreement, it is stated that the region will do [this task], but when nothing happens, it complicates things. (Participant 4)*

#### 3.1.3. Diverse Management 

The participants also described how they occasionally suffered from a lack of support when making decisions concerning new AT. They expressed frustration, as they felt that there was no consensus among different municipalities in Sweden regarding procedures and guidelines, leaving managers free to form their own. For example, the participants indicated that they had addressed this problem by reaching out to Swedish universities and decision-makers in other regions to gain decision-making support. According to the participants, these shortcomings on the part of the municipalities led to diverse management and ad hoc work procedures.

*Each municipality has different agreements, for example, that one prescribes. Thus, within the joint committee, there are various agreements and product range within each municipality. In the case of large procurement, the procurement is jointly made with the municipal association (sv. kommunförbundet). It is up to every municipality. There is often an assistive technology council, for example in the [city], it is the responsible occupational therapist who, until then with the unit manager decide and have the responsibility of the budget. If you decide that, this person should have this assistive technology or this more expensive assistive technology, and then go outside the product range, it is then up to every municipality [to decide]. We have nothing to say about it. […] We in Sweden do things differently from each other. We have agreements with suppliers about different and certain products. (Participant 20)*

### 3.2. Technology Aspects

The second subtheme, Technology aspects, included two codes: cost and function and sustainability and function. Within this subtheme, there were blurred boundaries between the subjects discussed, and therefore the subject of the functionality of AT was categorised within more than one code. The code cost and function included the cost estimations related to the functionality of the AT. More precisely, the code focused on which cost calculations were made, how they were made, and what tools and models were used (e.g., criteria in cooperation with others) by the managers to support decision-making. The code sustainability and function focused on the mangers’ aim of buying sustainable AT. The code includes the sustainability concerns (e.g., quality, maintenance, reusability, and replacement) that managers experienced in their decision-making. Each code will be discussed further below.

#### 3.2.1. Cost and Functions 

The participants reported that decision-making revolved around factors such as cost. They discussed performing economic health evaluations and their experiences with conducting cost–benefit analyses when considering new AT.

*We have a weighting if it is the price or function [that should be focus]. For example, we weigh if the price is 75% or the function should have 25% or another factor should have 100% weight during the procurement work. (Participant* 15)

*We do our own cost calculation, but it is quite poor. […] (Participant 21)*

*We have to calculate how many in the target group there are in the region. Replacing the [assistive device] is necessary, and it is a massive cost if the [assistive device] is not reusable. […] We have not had these new and big questions before. We have to understand the problem and think through the decision.*

*There is a calculation spin (sv. räknesnurra) where you look at how big the use of the [new assistive technology] is, what it would cost, what the criteria are. (Participant 16)*

#### 3.2.2. Sustainability and Function 

The participants highlighted aspects of sustainability and explained that they followed the national sustainability policy. Working towards becoming a more sustainable organization, the managers were aware of the impact of their purchases and aimed to invest in sustainable AT. Thus, the participants described how they focused on purchasing quality AT that lasted longer and allowed them to make purchases less frequently, thereby preserving environmental resources and reducing the volume of discard material. They stated that most of the organization’s AT was reconditioned, but they were occasionally forced to disassemble the AT for reuse or discard it. Additionally, some of the participants even expressed that it was much easier to discard than reuse some specific AT. Moreover, while the participants were aware of the limited global environmental resources, many of them indicated that they wished that they could achieve the environmental goal more successfully; however, they experienced challenges when working towards it. Another example of this challenge was with digital technology. The participants reported that the standard AT assortments, such as wheelchairs and walkers, are easier to reuse than more advanced digital technologies, which only work for a limited time and are rarely compatible with new gadgets, accessories, or products. The participants were aware of this issue, but, as according to them, there was no sustainability thinking in the digital technology area.

*We should have thought [the decision] through—that it is a sustainable product, that the life cycle will work, how long will this product last and yes, of course, the demand. If no one wants the product, it will not work. (Participant 2)*

*[When it comes to sustainability], we look at the whole picture when we take in assistive technology. How long we will be able to use it, the scrapping time, the ability to recondition the assistive technology, and the knowledge and skills of the technicians. Should we have it on stock or individual items? What are the lead times when ordering? These [questions] are essential when we are going to reuse assistive technology. It is much easier if we do not reuse. (Participant 18)*

*We are continually reviewing our routines about reusing and disposal of our used assistive technologies. Just a year ago, we did a great job of how long we can count on having a specific assistive technology in circulation. We looked at estimated life and set up some criteria for it, and we made some financial calculations. Repair of assistive technology must not cost more than a certain percentage of purchasing a new one. That part is crucial. (Participant 12)*

### 3.3. Patient Aspects 

The third subtheme, patient aspects, included the following three codes: legal rights, user perspective, and access to new technology. The first code, legal rights, included managers’ considerations about patients’ equality and equity. That is, all patients, despite their differences, were to be given the same opportunity to participate in daily activities and were included as a part of the society. The second code, user perspective, focused on managers’ consideration of the end-user’s usage of AT. The third code, access to new technology, related to managers’ consideration of patients’ access to AT. 

#### 3.3.1. Legal Rights 

All participants stressed the importance of acknowledging patients’ legal rights and considering their equality and equity. However, the participants expressed that how patients could draw upon their legal rights depended on the AT organization and its available assortment. Several of the participants said that they had a wide assortment of AT and that the AT users (i.e., patients) have the right to be part of the process of AT procurement.

*We have a large assortment, and as a patient, you are allowed to be part of the process. I think it needs to be customised as long it is possible. (Participant 12)*

For example, one of them explained that, in contrast to 20 years ago, patients today could choose between 14 different walkers instead of two as previously. Thus, the AT assortment has grown over the years, leading to a greater opportunity to be able to offer AT according to patients’ needs and preferences.

*Active in everyday life to be independence. Relieve the home care service or perform more activities self. There is probably more [patient] involvement in the selection of the actual product. In terms of procurement, we are procuring broadly to have a large product range to choose from. […] You give the patient their legal rights in that way. (Participant 23)*

*I think it is the basis for everything. We should do this so that everyone can be involved in society. A person should be able to get a wheelchair if the person needs one. We need to do this so that those who need hearing aids can become more involved. It is about access to sign language interpreters when a person cannot take part in the written and spoken information in the community. Vision aids should be provided so that those persons can see. To be able to increase the quality of daily life to be able to be part of life. That is a natural life. (Participant 17)*

Furthermore, the participants explained that, while they as managers acknowledge the importance of patients’ equality and equity, the prescriber actually meets the patient and through discussions with the patient, ensures that individual patient rights are respected.

*Our prescribers do this. To ensure patient involvement, it is part of the role of the prescriber’s role. We do not have a user organization (Swedish: Brukarorganisation), but that is something the consultants and we would use as suggestions for improvement. (Participant 24)*

*We have ongoing discussion, but we do not have any evaluation about [patient involvement]; it is much about the individual prescriber. (Participant 3)*

Additionally, the participants also stressed that there were challenges concerning patients’ legal rights to participate in AT procurement. According to the participants, patients are aware of their legal rights, and some of them use their knowledge about their rights to influence the prescriber when the prescriber evaluates the patient’s need for AT.

*Sometimes, the prescriber needs to limit the supply for the patient since [the wide assortment] makes it harder for the patient to choose. (Participant 10)*

*The regions should review the need-based regulations. A few years ago, when different areas had to undergo substantial [financial] cuts, we saw that it was essential that there were need-based regulations that were open and transparent. The kindest prescriber should not become persuaded [by the patient]. However, sometimes it is difficult to resist. There are those who scream loud. People [threaten] to go to the media. (Participant 7)*

#### 3.3.2. User Perspective 

According to the participants, most of the AT organizations carry out patient evaluations. These may be done at the management level in the AT organization or at the individual level when a consultant–patient meeting takes place. The participants stressed that the best feedback came directly from patients when they had meetings with the prescriber. Some of the participants described that they, as managers, had also tried to involve user organizations. However, the outcome of this collaboration was not always satisfying. The participants explained that, although the user organization had enthusiastic stakeholders, they communicated their options and ideas from a subjective- and personal perspective rather than from a collective and group level.

*We meet many users in different situations, but it is with mixed results. They must understand that they are not there in private, but as a group. They must understand that they represent the group, and this is difficult. It is difficult to get this objective perspective. (Participant 13)*

*We use a patient organization if we are going to procure something new. However, there is not a lot of activity around what the [patient] population wants. Yes, we have on a few occasions involved patient organizations in the [assistive device] group’s work, but it is probably the only occasion that I can say that we listened to that group. (Participant 1)*

*You should have focus groups, but then you end up having the user talking about their own situation or relatives talking about their old uncle. They just see their own situations and their needs. It is difficult to raise above your own situation. They only see their situation and only themselves and his or hers need. It is hard to get a good focus group, but we are thinking about how we can improve. (Participant 7)*

#### 3.3.3. Access to New Technology 

The participants stressed that it was important that patients were able to access AT according to their needs so that they could be independent and participate in society. However, they explained that this was a balancing act between several considerations, such as person-centred AT assessment, the size of the target group, and the ability to afford procurement of the desired AT. These considerations raised an additional question among the participants: how far their responsibility extends to provide patients with access to AT that would give them the opportunity to participate in leisure activities or perform their hobbies.

*We talked about these concerns a lot when prescribing. The supply planning has now changed, and it is to be digital. We have opened up about what kind of assistive technology we have; we have reference groups consisting of users. We did not have it at all before. Everything has passed through the prescribers. Before there were prescribers who judged and had thoughts. Back then, we did no investigations at all, and it was the prescriber who made the decisions. Now we opened up the process to be more person-centred. When can we say this assistive technology [will be procured] at this volume? The target group may consist of five people, but for these people, this [technology] is essential. How did we think earlier in the prescriber process? The discussion back then was that if it was such a small target group, it was not worthwhile to procure the assistive technology. These discussions made me scared of the dark! Who has the [power] to decide that? We do not have the right to control the target group and to claim that it must be a specific volume to be an AT [for us to buy]. It is not for us to decide this. (Participant 2)*

*Maybe not in the final decision and [the patients] have not been in the discussion: It happens at the level before. I think when all of the concepts are addressed. At the same time, what is our level of ambition and how much money do we have? What considerations do we take into account? [The patient] have to be able to handle themselves in everyday life, but if we think of assistive technology to be used at spare times and for hobbies, it is then [the patients] own responsibility? We have addressed the need that exists. (Participant 23)*

### 3.4. Knowledge Discrepancy 

The fourth subtheme, Knowledge discrepancy, emerged when the participants talked about the rapid development of new technology within the society that could be implemented in health care. The subtheme contained three codes. The first code, competence discrepancy, focuses on the competencies that the managers perceived the AT organizations were lacking and needed to develop in order to handle new and improved technology. The second code, knowledge sharing, related to how managers share knowledge with other managers and AT organizations. The last code, supplier dependency, revolved around the dependency that exists between suppliers and the AT organization as well as the role the suppliers play in providing the AT organization with knowledge about the new and procured AT. 

#### 3.4.1. Competence Discrepancy

The majority of the participants indicated that their AT organizations possessed adequate competence for the existing technology. However, they stressed that this was on a general level and that they had noticed that some employees, such as front-line personnel who met patients did not have the required technology competence. According to the participants, they saw a need to provide personnel who lack technology competence with the opportunity to learn about and use technology. However, a problem highlighted by the participants is the fact that employees already experience a high workload, and thus adding technology development initiatives would increase the stress employees already face in their jobs. Additionally, some participants argued that individual incompetence was absolved by age and that younger employees had more interest in developing their competence.

*I can say that I can sometimes feel, about digitalization, that we do not have the desirable competence in our employees. It would feel a little calmer if there were professionals who could make scientific assessments of the value of different assistive technology devices. We have many of our employees who do not have sufficient expertise to help. It is a shortcoming today that we still have people born in the 50′s and 60′s. We cannot use the system. We are not born with it, and we cannot use the system. We do not dare as much. How should we be able to prescribe such [digital] devices then? (Participant 7)*

*I think we will acquire the knowledge when new assistive technology devices come, that we will then develop skills. It is right now manageable, though not on the prescriber’s side where it is heavy. It is more about people’s interest. It can be a generation issue, who has grown up with digitalization or not […] (Participant 8)*

*I think we are already very far ahead with the technology that already exists. We are not that far behind. Well, I think we have the competence. The technology is good, and we have [assistive technology devices] that are already advanced. (Participant 23)*

*If we were going to handle more of the digital technology, we would need to bring in more expertise and develop expertise in the area. […] Unfortunately, this is the current state of our organization. I must say that within the municipality, there is no interest or no knowledge in the area. (Participant 8)*

#### 3.4.2. Knowledge Sharing 

All of the participants mentioned that they perceived challenges within their positions. They requested an evidence-based perspective and scientific evidence that they could draw upon in their decision-making; for example, they need evidence showing that AT is working according to its purpose and scientific assessment of the value of different AT. Other requests were to increase knowledge sharing among the different AT organizations and to create transparent and national governmental directives for AT organizations. The participants explained that there was considerable confusion about responsibility lines, that is, who had the responsibility (e.g., the region or the municipality) to carry out certain decisions and tasks, leaving patients caught in the middle. While some participants stressed that they were networking with other managers, they had different contexts and structures to relate to, which made knowledge sharing complicated.

*We are talking about having an Open api that must be a common solution [for all AT organizations] and a shared vision for our region, or preferably in the whole Sweden. (Participant 6)*

*This is difficult because a patient can get assistive technology from [the region]. However, the municipalities do not give them the same [assistive technology]. They consider other [decision] factors and have different criteria they use to grant patients assistive technologies. Therefore, it can be that way also. It is not certain that the directions and agreement follow those used by the region. Then, it can differ between municipalities. It is a question about being fair, then by doing differently, it can be unfair [for patients]. Sweden should regulate on a national level, so there are no big differences in the country. (Participant 12)*

*We can say that [a specific assistive technology] is the responsibility of our region [to provide]. However, we have seen that our municipality lacks this assistive technology. Therefore, our municipality allows us to cross this product range boundary because we do not have proper control mechanisms. However, in other municipalities, they are not allowed to cross the product boundaries because it is the regional responsibility. Here, we have a problem. For where the region does not acquire this assistive technology, these users will be without. Therefore, there is that kind of problem. Yes, we really wish for more overall governmental and national directives. We really should have those. (Participant 7)*

*We have understood that we need to work together and work with changes based on assistive technology organizations, organization development. We need a united approach so that we see that the management has realised this. (Participant 23)*

#### 3.4.3. Supplier Dependency 

The participants reported that they had suppliers of AT. There were agreements between the supplier and the AT organizations about AT procurements. These agreements focused on repeated and standardised AT procurement. The suppliers present newly developed AT available on the market. The participants stated that the suppliers played an important role in educating the AT organizations about new available technologies and how to use them. They also said that they would attend AT courses supervised by the suppliers. However, this created a supplier dependency. The participants indicated that it was not easy to object to and critically analyse the suppliers’ offerings and thus requested a more evidence-based approach to the evaluation of new AT.

*When it is about what is procured and agreements exist with the suppliers, they do what we want and say. Then, they have product displays and can present what products are new. However, it is, after all, the existing major suppliers. We have assortment groups that we use to see what is going on and what new assistive technology will soon make an entrance. (Participant 1)*

*We educate our technicians and prescribers with supplier courses. We increase their knowledge and skills with courses provided by the suppliers. (Participant 13)*

*Often, the supplier will present evidence. It does not feel so good. We want [the evidence] to be based on the function and the need, using a more objectively perspective. (Participant 6).*

## 4. Discussion 

From the analysis, an overall theme emerged: decision-making is in the making. This theme refers to the on-going decision-making work that managers undertake when a new technology is being considered for procurement by the AT organization. The data show that managers in AT organizations usually focused on the standardised part of the AT assortment of the AT organization. However, some of the decisions were made ad hoc, as they involved unrepeated and unique procurement of new AT to complement the standardised AT assortment. To understand what aspects influence decision-makers’ decisions when new technology is being considered for procurement by an AT organization, we identified four subthemes: supportive aspects, technology aspects, patient aspects, and knowledge aspects.

The data revealed that managers experience uncertainty when making ad hoc decisions about new AT procurement, and thus the unstandardised part of their AT assortment. One reason for this was the supportive aspects. The managers experienced a lack of decision support, such as formal national policies and guidelines on making decisions about new AT. A majority of the managers stressed that they had, on their own initiative, attempted to identify, review, and develop policies and guidelines to facilitate their decision-making regarding new AT. It was also difficult for the managers to make decisions about the technology aspects, especially analysing and interpreting information about cost, sustainability, and functionality, as well as weighing these different variables and deciding upon a suitable balance between them.

Furthermore, the data also highlighted that the managers acknowledged the patient aspects, such as equality and equity. The managers considered AT a powerful way for patients to be included as part of society, and therefore they considered patients’ needs, preferences, and AT usage in their work. These findings are in line with those of previous research [43,44,45], which argues that AT is important for the maintenance of patients’ autonomy, participation, and communication in society.

Thus, the managers followed a person-centred approach, striving to involve user organizations (i.e., patient organizations) to make patients’ voices audible in the decision-making. This work to involve user organizations was portrayed as challenging, as the managers interpreted the “voices” they heard as subjective and individual-centred. However, the value of these voices is important, since there is a risk that AT could disable older adults if their goals, desires, capabilities, and social context are not taken into consideration [22,46]. Moreover, as Kielhofner [44] stresses in MOHO, since people differ in their abilities and beliefs about their own abilities, a specific environment can present several hindrances to one person while not affecting another. This implies that the ability to manage the usage of AT is also important for older adults.

Additionally, the managers raised questions about patients’ access to new AT and how the differences in AT assortment might limit their opportunities to access AT in an equal and equitable manner. In fact, the data indicate that the managers were concerned about how knowledge discrepancy aspects could influence their decision-making regarding future AT assortments. According to the managers, there is a need to develop technology competence to make future decisions about innovative technology procurement and to provide innovative technologies to patients. To address the issue of a lack of competence, the managers emphasised the need to have resources to allow employees time and space for competence development and to create an interest in new technology. The data revealed that suppliers play an important role in increasing the competence of AT organizations in relation to new AT market introductions and how to use new AT. Managers faced a challenge in balancing the power and dependency that exist between suppliers and AT organizations due to the knowledge advantage of the suppliers. In line with this development, the managers raised concerns about the introduction of innovative technology. While they saw the potential in advanced technology from a patient’s perspective, they were concerned about knowledge sharing among AT organizations in Sweden and requested a national evidence-based approach to tackle the challenges in AT decision-making. 

Notably, decision-making in organizations is a well-studied field of research where the focus is on how to improve organizational decisions, e.g., [47,48,49,50]. Prior studies on decision-making in organizations have found that decision-makers seek to formulate various alternatives while considering internal and external constraints, cf., [47,48,49,50]. The focus of the decision-makers is to understand the various alternatives that exist, to predict their end results, and to select the best alternative [51]. Our findings resemble these prior findings, where the managers in the AT organizations shared the same focus on making “good” decisions while considering, for example, financial, technology and knowledge constraints. According to Rousseau [52], an evidence-based approach may improve the quality of decisions in organizations. This supports the AT organizations’ requests for an evidence-based approach to facilitate AT decision-making.

Finally, the specific professional context of this study provides valuable insights into decision-making in AT organizations. Both theoretical and empirical research insights were derived from the analysis. Specifically, we were able to gain insights into how and in which limiting or facilitative conditions decision-makers experienced their roles in these organizations. However, there were some limitations due to the design of the study. Although this study was based on interviews with decision-makers from a national network of AT managers, it might not capture all possible decision-making experiences, and the questions might not have been broad enough to capture all possible details. Furthermore, the backgrounds or experiences of the decision-makers were not explored or reflected upon in this study. This means that the way in which managers draw upon past experience was not addressed in this study. 

Based on the results of this study, we argue that there is a need for future research on AT decision-making to further develop the research field. One suggestion for future research is the development of comprehensive, easy-to-use tools for decision-making based on, for example, ICF and ISO 9999 as common frameworks applicable to the Scandinavian (and European) context. Another suggestion for future research is to study different stakeholders’ perspectives on AT decision-making. The analysis shows that managers rely on the engagement of stakeholders (e.g., patients, relatives, staff, suppliers, other AT organizations, municipalities, government) when making decisions about AT procurement. Therefore, and inspired by the work of Freeman [53], it would be fruitful for future research to study the interconnection between stakeholders, their aligning interests, and communication to improve AT decision-making. 

## 5. Conclusions

We sought to contribute to the knowledge on AT, especially on AT decision-making, by identifying aspects that influence decision-makers when new technologies are being considered for procurement by an AT organization. Our study concludes that supportive aspects, technology aspects, patient aspects, and knowledge aspects influence AT decision-making. AT has been shown to be a successful tool for supporting older adults to age in place [4], as its design supports people with diverse functional abilities [6]. With such a tool, it is crucial that patients are in focus and that all patients are treated equally and have the same opportunity to access AT irrespective of where they live. This is a legal right that patients have in Sweden. Moreover, our study reveals that there are challenges related to AT decision-making, as decision-makers aim for a person-centred approach that might lead to better outcomes and reduce the risk of AT abandonment. One challenge is the general lack of evidence-based support in AT decision-making. Another challenge is the lack of national support in the form of national guidelines and policies, which was also noted in a new report [54]. Increased communication between government, regions, and municipalities to support the development of national guidelines and policies would likely enhance the possibility of reaching the goal of person-centred care. Notably, AT organizations are governed by local political councils, which add to the complexity since the resource frames of the AT organizations change each fourth year with the elected council. Collaboration between AT organizations might also contribute to decreasing current ad hoc and diverse AT decision-making. In sum, AT decision-making is a complex task that requires the inclusion of person-centred and evidence-based perspectives as well as the supportive engagement of stakeholders.

## Figures and Tables

**Table 1:** Emergent themes, subthemes, and codes.

Theme	Decision-Making Is in the Making										
Sub-Themes	Supportive Aspects			Technology Aspects		Patient Aspects			Knowledge Aspects		
Codes	Policies and guidelines	Development initiatives	Diverse management	Cost and function	Sustainability and function	Legal rights	User perspective	Access to new technology	Competence discrepancy	Knowledge sharing	Supplier dependency
